# Natural Bacterial and Fungal Peptides as a Promising Treatment to Defeat Lung Cancer Cells

**DOI:** 10.3390/molecules28114381

**Published:** 2023-05-27

**Authors:** Kamila Rybczyńska-Tkaczyk, Anna Grenda, Anna Jakubczyk, Paweł Krawczyk

**Affiliations:** 1 Department of Environmental Microbiology, The University of Life Sciences, Leszczyńskiego Street 7, 20-069 Lublin, Poland; kamila.rybczynska-tkaczyk@up.lublin.pl; 2 Department of Pneumonology, Oncology and Allergology, Medical University of Lublin, Jaczewskiego Street 8, 20-954 Lublin, Poland; pawel.krawczyk@umlub.pl; 3 Department of Biochemistry and Food Chemistry, University of Life Sciences in Lublin, Skromna Street 8, 20-704 Lublin, Poland; anna.jakubczyk@up.lublin.pl

**Keywords:** peptides, bacteriocins, fungal metabolites, lung cancer

## Abstract

Despite the increasing availability of modern treatments, including personalized therapies, there is a strong need to search for new drugs that will be effective in the fight against cancer. The chemotherapeutics currently available to oncologists do not always yield satisfactory outcomes when used in systemic treatments, and patients experience burdensome side effects during their application. In the era of personalized therapies, doctors caring for non-small cell lung cancer (NSCLC) patients have been given a powerful weapon, namely molecularly targeted therapies and immunotherapies. They can be used when genetic variants of the disease qualifying for therapy are diagnosed. These therapies have contributed to the extension of the overall survival time in patients. Nevertheless, effective treatment may be hindered in the case of clonal selection of tumor cells with acquired resistance mutations. The state-of-the-art therapy currently used in NSCLC patients is immunotherapy targeting the immune checkpoints. Although it is effective, some patients have been observed to develop resistance to immunotherapy, but its cause is still unknown. Personalized therapies extend the lifespan and time to cancer progression in patients, but only those with a confirmed marker qualifying for the treatment (gene mutations/rearrangements or PD-L1 expression on tumor cells) can benefit from these therapies. They also cause less burdensome side effects than chemotherapy. The article is focused on compounds that can be used in oncology and produce as few side effects as possible. The search for compounds of natural origin, e.g., plants, bacteria, or fungi, exhibiting anticancer properties seems to be a good solution. This article is a literature review of research on compounds of natural origin that can potentially be used as part of NSCLC therapies.

## 1. Introduction

According to the Globocan database, the estimated global number of new cases of cancer in 2020 in both sexes and all ages was 19,292,789. The estimated mortality due to cancer worldwide in the same year was 9,958,133 [1]. It is estimated that the number of new cases of cancer will have increased to about 30 million and the death rate will have risen to about 16 million cases by 2040. These statistics indicate that, despite the increasing availability of modern therapies, including personalized therapies (small molecule therapy and immunotherapy), there is a strong need to search for new drugs that will be effective in the fight against cancer. Plant substances or plant-based chemotherapeutics have become one of the most important elements of the systemic treatment of cancer patients. For example, paclitaxel is the best-known plant-derived chemotherapeutic agent from *Taxomyces andreanae*. It is extracted from the inner bark of *Taxus brevifolia* and is used in the treatment of lung, ovarian, and breast cancer [2]. Microbes are the other natural sources of anticancer substances. Antibiotics derived from microorganisms exert the following effects: alkylation and thus damage to the DNA of cancer cells (melphalan), inhibition of DNA replication (mitomycin), or interruption of cell mitosis (doxorubicin). These drugs are widely used in systemic anticancer treatment. Currently, clinicians can use modern molecularly targeted therapies or immune checkpoint-targeting therapies. In the former type, specific gene alterations predisposing a patient to inclusion in treatment with inhibitors of kinases involved in cellular signal transduction pathways are searched for in BRAF (B-Raf Proto-Oncogene, Serine/Threonine Kinase), MEK (Mitogen-Activated Protein Kinase Kinase 1), RAS (RAS Proto-Oncogene, GTPase), EGFR (Epidermal Growth Factor Receptor), RET (RET Proto-Oncogene), HER2 (Erb-B2 Receptor Tyrosine Kinase 2), ROS1 (ROS Proto-Oncogene 1, Receptor Tyrosine Kinase), ALK (Anaplastic Lymphoma Receptor Tyrosine Kinase), and NTRK (Neurotrophic Receptor Tyrosine Kinase). Depending on the type of cancer, different percentages of patients are diagnosed with genetic variants. Activating *BRAF* mutations are observed in approximately 50% of melanoma patients [3]. In these patients, the identification of *BRAF* alterations, in particular, the V600E variant, allows the use of BRAF inhibitors: vemurafenib, dabrafenib, or encorafenib and MEK inhibitors: cobimetinib, tremetnib, or binimetinib [3]. Activating genetic variants in the *EGFR* gene, qualifying for treatment with *EGFR* inhibitors (erlotinib, gefitinib, afatinib, dacomitinib, and osimertinib) are observed in 10–15% of Caucasian patients and in at least 40% of Asian non-small cell lung cancer (NSCLC) patients [4]. In turn, *ALK* and *ROS1* rearrangements are detected in approximately 4.5% and 1% of non-small cell lung cancer patients, respectively [5,6]. In the presence of *ALK* driver alterations, ALK tyrosine kinase inhibitors (crizotinib, ceritinib, alectinib, brigatinib, ensartinib, and lorlatinib) may be used. In turn, the crizotinib or entrectinib can be used in patients with *ROS1*-positive NSCLC. Molecularly targeted treatment has also shifted towards ‘tissue agnostic’ drug treatment [7]. The assumption is based on a model where it does not matter in which type of cancer a given genetic variant occurs but on the fact that the treatment targeted at the protein product resulting from a given mutation is effective. This approach is highly beneficial when genetic variants are detected in a very small percentage of patients with different types of cancer, as is the case of *ROS1* or *NTRK1/2/3* rearrangements.

These percentages indicate that not all cancer patients can be subjected to targeted therapy. Another therapeutic approach is the immune checkpoint-targeting immunotherapy. The use of monoclonal antibodies targeted the PD-1 (Programmed Cell Death 1), PD-L1 (Programmed Cell Death 1 Ligand 1), or CTLA4 (Cytotoxic T-Lymphocyte Associated Protein 4) molecules has revolutionized the treatment of oncological patients, bringing significant benefits, i.e., an extension of survival time or improvement of the quality of patient’s life related to the absence of side effects such as those occurring in chemotherapy treatment [8,9]. This does not mean that the therapy has no flaws. Side effects may occur, but they are different than those induced by TKI inhibitors or chemotherapy. In addition, resistance to immunotherapies may be observed, but its causes are not fully understood [10]. Several mechanisms of resistance to immunotherapies have been identified, such as the absence of tumor antigens, defective antigen presentation, modulation of critical cellular pathways, epigenetic changes, and changes in the tumor microenvironment. The elucidation of these mechanisms has contributed to the implementation of the chemoimmunotherapy strategy, where chemotherapy results in the presentation of neoantigens on cancer cells, making them more visible to the immune system recognizing them by blocking the PD1/PD-L1 or CTLA4/B.7 pathways. The estimated percentage of US patients with cancer who are eligible for checkpoint inhibitor drugs in 2018 was 43.63%. The percentage of patients estimated to respond to ICI in 2018 was 12.46% [11].

All this indicates that chemotherapy will not be replaced by modern therapies with kinase inhibitors or immunotherapy but will still be an important element of the strategy of treatment in oncological patients. It should also be mentioned that chemotherapy is important as an adjuvant or neoadjuvant therapy. According to the ACS (American Cancer Society), in the USA, 56% of women with stage III breast cancer undergo surgical removal of the breast followed by chemotherapy [11]. As reported by the ALA (American Lung Association), over 20% of lung cancer patients are treated surgically. In addition, chemotherapy in combination with immunotherapy is still the only option for small cell lung cancer. Moreover, according to the NCI (National Cancer Institute), on average, 64.5% of patients (age range 20–80 years old) with stage IIIB or IV non-small cell lung cancer in 1996–2018 received some chemotherapy regiments. All this indicates that chemotherapy is still needed, and the search for new natural sources of chemotherapeutics may contribute to improvement of the effectiveness of treatment of oncological patients, including those with lung cancer, which ranks second in terms of the overall incidence in the world and first in terms of the cause of cancer-related death. The search for plant, bacterial, or fungal compounds seems to be a challenge that can improve chemotherapy outcomes. There are some papers on this topic, but still in vitro, in vivo, and clinical research is required [12,13,14].

## 2. Anticancer Peptides (ACPs)

One of the potential innovative therapeutic methods in the fight against cancer cells is the use of anticancer peptides (ACPs). Their properties are determined by the amino acid composition and the addition of functional groups, which affects their conformation, charge, stability, bioavailability, more efficient tumor/tissue penetration than antibodies, and effectiveness. ACPs are usually composed of 10–60 amino acid residues that can inhibit the migration and proliferation of tumor cells or inhibit the formation of tumor vasculature. The advantage of ACPs is their lower ability to cause drug resistance. On the other hand, they can be hydrolyzed by proteases or cause cytotoxicity [15]. These compounds are either natural or modified with positive cationic moieties or functional groups to enhance their therapeutic efficiency and reduce side effects. Additionally, peptides can be modified to attack tumor cells and prevent tumor progression [16]. They can be used in a variety of ways to treat cancer. Anticancer peptides can be applied as drugs or can deliver drugs to cancer cells. They also simulate natural proteins to enhance or inhibit signal transduction and mediate therapeutic transport across the barrier [16,17]. Peptides with anticancer properties can be synthesized in various cells and conjugated with other compounds and subfields in various ways. Some peptides can cross the blood–brain barrier and affect the central nervous system. This property seems to be extremely important in terms of the appearance of metastases in the head area in the advanced stages of lung cancer. The advantage of bioactive peptides is that their production is simpler than the production of proteins and antibodies. In addition to their simplicity and ease of transport, peptides have a longer shelf life [17]. Cancer cells differ from healthy cells in their membrane. The anticancer effect of peptides is based on the induction of cancer cell apoptosis and necrosis through membrane lysis or the formation of pores in the membrane [18]. Cholesterol is a component of the cell membrane protecting healthy cells from the lytic action of various factors by changing the fluidity of the cell membrane. Cancer cells exhibit higher fluidity of the cell membrane than healthy cells. Their more abundant microvilli increase their surface area. In addition, healthy cells are electrically neutral, while cancer cells contain a negatively charged component on their surface, which leads to membrane destabilization, cytotoxicity, and cell lysis during interactions with small molecules, e.g., ACPs [19]. The interactions between peptides and the healthy cell membrane are hydrophobic, while electrostatic interactions occur between peptides and the membrane of tumor cells [20]. Currently, extensive research is being conducted on the use of peptides in the treatment and diagnosis of many cancers to develop peptide therapy. The basis of this therapy is the use of selective targeting of tumor-specific receptors. Peptides that target the tumor are more efficient in the penetration of tumor tissues than antibodies. Additionally, chemical modifications can increase their stability and pharmacokinetics. Peptides have already been applied in diagnostics, cancer imaging, and targeted drug delivery [17].

### 2.1. Classification and Mechanism of Action of ACPs

Anticancer peptides are characterized by different structures, modes of action, selectivity, and efficacy against specific cancer cells. There are many ACPs derived from different species and characterized by different properties; hence, these compounds can be classified in different ways. The most common classification based on their structure has distinguished four categories of ACPs: α-helical, β-pleated sheets, random coil, and cyclic [15]. 

It has been shown that the hydrophobic amino acid residues in the alpha structure enhance their cationic properties, while the amphipathic properties of these structures play an important role in cytotoxicity against cancer cells [21]. The formation of disulfide bridges is necessary to maintain the β-pleated sheet structure. It is worth noting that ACPs with an α-helical structure have higher antitumor properties than β-pleated peptides and higher toxicity to normal tissue [15].

Another classification is based on the mechanism of action: molecularly targeted peptides, which directly act on cancer cells via cytotoxic, antiproliferative, and apoptotic activities, ‘guiding missile’ peptides, or binding peptides, which are drug-binding peptides used for transporting drugs into cancer cell targets, and cell-stimulating peptides that indirectly influence other stimulating cells to kill cancer cells, e.g., via immunomodulatory activities and hormone receptors [22,23].

### 2.2. Effect of Peptides on Lung Cancer Cells

Many studies indicate the possibility of using peptides in the treatment of lung cancer due to their size and properties. These peptides can be obtained from food and non-food protein or through synthesis, as shown by results of in vivo or in vitro studies. It was noted that tryptophan-containing peptides exhibited increased cytotoxicity against non-small cell lung adenocarcinoma A549 cells, and their hemolytic activity was found to increase as well [24].

A protein obtained from *Porphyra haitanesis* was shown to be a natural source of anticancer peptides. To obtain bioactive peptides, the protein was hydrolyzed using trypsin, and then the peptides were purified with the use of chromatographic techniques. Three peptides showing antiproliferative properties against five cancer cell lines: MCF-7, HepG-2, SGC-7901, A549, and HT-29 were isolated and their IC_50_ (half maximal inhibitory concentration) values were in the range from 191.61 to 316.95 μg/mL [25]. Wang and Zhang described a new peptide YGFVMPRSGLWFR obtained from papain hydrolysates of *Spirulina* (*Arthrospira*) *platensis* proteins, which exerted high inhibition activity against A549 cancer cells with the IC_50_ value of 104.05 μg/mL [26].

However, the exact relationship between the structure and action of peptides has not been thoroughly clarified. It is known that the presence of certain amino acid residues enhances the anticancer efficacy of peptides. To elucidate the relationship between the structure and activity of peptides, their synthetic analogs are being studied. Thus, an analog of alyteserin-2a (ILGKLLSTAAGLLSNL.NH2), i.e., a peptide isolated from the skin secretion of the midwife toad *Alytes obstetricians*, was studied. In the analog, the amino acids on the hydrophobic side of the helix were replaced by L-tryptophan and the hydrophilic amino acids were substituted by one or more L-lysine or D-lysine residues. The results indicated that the tryptophan-containing peptides had up to 11-fold higher cytotoxic activity against non-small cell lung adenocarcinoma cells A549, and their hemolytic activity against human erythrocytes increased as well. The activity of the N15K analog on A549 cells (LC50 = 13 µM) increased sixfold relative to alliterin-2a and the therapeutic index (LC50 ratio for erythrocytes and tumor cells) increased twofold. In contrast, the insertion of the 11 D-lysine residue in the N15K analog produced a peptide which retained potency against A549 cells (LC50 = 15 µM), with a 13-fold higher therapeutic index relative to the native peptide. Therefore, increasing hydrophobicity while maintaining amphipathicity via the insertion of the L-tryptophan peptide in the structure yielded a peptide with increased antitumor activity, compared to the starting peptide [24].

Patil and Kunda studied cationic antimicrobial peptide D-LAK-120A (KKLALALAKKWLALAKKLALALAKK-NH2) as an anticancer factor in various NSCLC cell lines [27]. Their results indicated cytotoxicity in concentrations between 4.0 and 5.5 µM against A549, H358, H1975, and HCC827 cell lines. An increase in lactate dehydrogenase (LDH) activity and propidium iodide (PI) uptake across the compromised membrane was suggested as an inhibition pathway. The study also demonstrated that D-LAK-120A was an inductor of lung cancer apoptosis and cell arrest in the S phase (DNA synthesis) of the cell cycle. Moreover, this peptide was shown to be involved in the in vitro inhibition of single-cell proliferation and cancer cell migration. The 3D spheroid indicated tumor reduction, which suggests the potential use of D-LAK-120A as an anticancer substance for non-small-cell lung cancer treatment and a potential therapeutic agent [27].

Lunasin, which consists of 43–44 amino acid residues with nine consecutive aspartic acids at the C-terminus, is a soybean peptide with indicated anticancer activity. It shows both chemopreventive and chemotherapeutic activities. Noteworthy, treatment of NSCLC cells with this peptide was limited by cell-line anti-proliferative effects on anchorage-dependent growth; on the other hand, two normal bronchial epithelial cell lines were unaffected. The study conducted in a murine model indicated that 30 mg lunasin/kg body weight per day decreased the NSCLC H1299 tumor volume by 63.0% on day 32. Moreover, this peptide inhibited cell cycle progression at the G1/S phase interface in the NSCLC H661 cells but did not induce apoptosis [28]. It was also shown that protein hydrolysates obtained from *Enteromorpha prolifera* may be a source of a peptide with anticancer properties. A heptapeptide with the amino acid sequence GPLGAGP exhibited potent antiproliferative activity toward several human cancer cell lines. The results of the study indicated that the IC_50_ values for NCI-H460, HepG2, and A549 were 0.3686, 1.2564, and 0.9867 mg/mL, respectively. This peptide-induced cell apoptosis in a dose-dependent manner [29].

Peptides are also used to support the action of anticancer drugs. Nanoconjugates of alendronate sodium (potent inhibitor of farnesyl pyrophosphate synthase) with mastopran (potent anticancer polypeptide isolated from wasp venom) (ALS-MP) were tested as potential compounds to be used in the inhibition of lung cancer. The results showed a lower IC_50_ value (1.3 ± 0.34 μM) compared to ALS (IC_50_ 37.6 ± 1.79 μM). Moreover, after treatment with the ALS-MP nanoconjugates, a higher percentage of cells in the G2-M phase was noted during cell cycle analysis [30]. Cisplatin is used as a standard and effective drug in lung cancer chemotherapy. To enhance the chemotherapeutic effects of cisplatin, the potential of a peptide from *Lentinus squarrosulus* (*Mont*.) used as an adjuvant was assessed. The results indicated that 24 h preincubation with 5 μg/mL of the peptide prior to treatment with 5 μM cisplatin significantly diminished % cell viability in various human lung cancer cells. However, it did not reduce the lifespan in the human dermal papilla and proximal renal cells. Flow cytometry indicated augmentation of cisplatin-induced apoptosis in the lung cancer cells. The peptide-pretreated lung cancer cells showed enhanced cisplatin-induced apoptosis and inhibition of colony formation. These data indicate the possibility of using a new combination therapy based on platinum compounds in the treatment of lung cancer [31].

## 3. Compounds of Bacterial Origin, Including Peptides, against Lung Cancer Cells

The anticancer activities of metabolites from many bacteria have been shown in recent years, but researchers are testing various niches that can be a source of bacterial anticancer substances. Examples of compounds of bacterial origin showing anticancer abilities against lung cancer cells are summarized in Table 1.

Silva et al. conducted their research on *Deschampsia antarctica* as a reservoir and source of microorganisms that produce metabolites with anticancer properties [32]. They performed next-generation sequencing with analysis of the 16S rRNA gene. They selected *Streptomyces* sp. CMAA 1527 and *Streptomyces* sp. CMAA 1653 as candidates for producing natural compounds with the potential to control the proliferation of breast (MCF-7), glioblastoma (U251), non-small cell lung (NCI-H460), and kidney (786-0) human cancer cell lines. The analyses indicated that Cinerubin B and actinomycin V were the predominant compounds identified in *Streptomyces* sp. CMAA 1527 and *Streptomyces* sp. CMAA 1653, respectively, which had anticancer properties [32]. Cinerubin B is an anthracycline antibiotic, but its importance as an anticancer agent has not yet been extensively studied. However, there are studies on actinomycin V which show cytotoxic activity and inhibition of proliferation, invasion, and migration of cancer cells, i.e., processes related to high expression of vimentin and E-cadherin [33,34]. As reported by Lin et al., actinomycin V caused up-regulation of p53, which suppressed the growth of lung cancer (A549) cells and induced cell cycle arrest and apoptosis. The cytotoxic activity of actinomycin V against the A549 cells with wild-type p53 was stronger than against NCI-H1299 cells (p53-deficient lung cancer cells). Actinomycin V decreased the expression of such M-phase related proteins as CdC2 (Cell Division Control Protein 2 Homolog), CdC25A (Cell Division Cycle 25A), and CCNB1 (Cyclin B1) arrested the cells in the G2/M phase, and subsequently triggered apoptosis by mediating the expression of the Bcl-2 family proteins (Bax and Bcl-2) [33]. Other results obtained by Lin et al. showed that actinomycin V treatment significantly down-regulated the levels of N-cadherin and vimentin expression, which promotes cellular proliferation, invasion, and migration of many cancer cells, including lung cancer lines [34]. Therefore, Actinomycin V may block the passage of EMT in cancer cells by reducing cell invasion and migration [34].

Sharma et al. studied the anticancer activity of enterocin from *Enterococcus faecium* 12a culture supernatant [35]. They found that enterocin 12a inhibited the growth of cell lines in a dose-dependent manner, and the 50% inhibitory concentration of this enterocin in lung cancer cell lines was 0.08 μg/mL [35]. The authors suggest that enterocin 12 should be tested in in vivo experiments as a potential anticancer agent. Arunmanee et al. tested the anticancer activity of colicin N [36]. Colicin N is a bacteriocin (peptide) produced by *Escherichia coli* that acts against other *E. coli* strains. It kills bacteria via the formation of pores in the cell membrane, leading to membrane depolarization and cell destruction. It can bind to receptors and induce formation of pores in the cell membrane. The authors found that treatment of human lung cancer H460, H292, and H23 cells with colicin N at 5–15 µM selectively caused cytotoxicity with no noticeable cell death in human dermal papilla cells [36]. In addition, in protein analysis, lung cancer cells cultured with colicin N (10–15 µM) for 12 h were found to activate extrinsic apoptosis [36]. This was evidenced by the reduction of c-FLIP (Caspase-Like Apoptosis Regulatory Protein) and caspase-8 as well as the altered expression of intrinsic apoptosis signaling proteins Bax (BCL2 Associated X, Apoptosis Regulator) and Mcl-1 (MCL1 Apoptosis Regulator, BCL2 Family Member), i.e., an anti-apoptosis protein overexpressed in NSCLC [36]. Furthermore, 5–15 µM colicin N downregulated the expression of activated AKT kinase (AKT Serine/Threonine Kinase 1) as well as integrins β1 and αV in human lung cancer cells. The authors conclude that colicin N exhibits selective anticancer activity associated with suppression of the integrin-modulated survival molecule. In a further study conducted by Arunmanee et al., it was shown that resurfacing of the receptor-binding domain of colicin N (solvent-exposed aspartic (D) and glutamic (E) acids of the receptor-binding domain substituted by lysine residues (K) as these substitutions, ColN^+12^) increased the net positive charge of the protein surface. ColN^+12^ was found to enhance its cytotoxic effect on human lung cancer cells [37]. The toxicity of ColN^+12^ was cancer selective. Human lung cancer cells H460 and H23 were sensitive to ColN, but human dermal papilla cells were not. ColN^+12^ also showed more potent toxicity against cancer cells than ColN^WT^. This confirmed that the polycationic resurfacing method improved the anticancer activity of ColN toward human lung cancer cells. The decrease in the percent cell viability determined was particularly evident in lung cancer cells exposed to 10 µM ColN^WT^ and 5–10 µM ColN^+12^. As shown by the comparison of the use of the peptides at the same concentrations, the treatment with ColN^+12^ induced a greater reduction of viable cells than the ColN^WT^ treatment in both H460 and H23 lung cancer cells. The determination of the mode of cell death demonstrated that the culture with either ColN^WT^ (10 µM) or ColN^+12^ (5–10 µM) induced apoptosis. Interestingly, the authors indicate that, although the bactericidal activity of the ColN mutant was reduced, its cytotoxicity against human lung cancer cells was significantly increased, and selectivity for lung cancer cells was noted [37].

Nisin ZP is a polycyclic antimicrobial peptide produced by the Gram-positive bacterium *Lactococcus lactis*. Investigation results indicated a concentration-dependent decline in NSCLC cell viability with an IC_50_ value of 132.4 μM in A549 cells and 137 μM in H1299 cells upon nisin ZP treatment [38]. The results revealed that nisin ZP induced selective toxicity in lung cancer cells, compared to healthy cell lines [38]. Nisin ZP induced apoptosis and cell cycle arrest in the G0/G1 phase in non-small cell lung cancer cells, irrespective of the p53 expression. The inhibition of proliferation was caused via non-membranolytic pathways by mitochondrial membrane depolarization and enhanced generation of reactive oxygen species. Furthermore, nisin ZP was able to reduce the clonal proliferation and migration of tumor cells, indicating its effect in advanced metastatic non-small cell lung cancer [38]. Bacteriocins, which are mostly peptides produced by bacteria, are considered antimicrobial substances; given their ability to disintegrate the cell membrane and destroy cells, they are widely studied as potential anticancer substances. Laterosporulin10 isolated from *Brevibacillus* sp. strain SKDU10 demonstrated cytotoxic properties against lung cancer H1299 cells at a concentration below 10 μM [39]. It has also been observed that this peptide can induce apoptosis and necrosis [39]. *Bacillus amyloliquefaciens* BTSS3 produces glycine-rich bacteriocin BaCf3, i.e., a hydrophobic peptide with a characteristic property of AMPs acting on the cell wall [40]. The structure of BaCf3 obtained from TrRosetta had antiparallel β-sheets resembling laterosporulin [40]. In silico studies with an anticancer target have proved that bacteriocin BaCf3 is a potential anticancer drug candidate. Saidumohamed et al. also performed in vitro tests on a lung cancer cell line (A549) and found that the rate of proliferation of cells treated with BaCf3 was higher after 48 h incubation but lower than 50% after 72 h incubation, indicating that it is a slowly acting anticancer compound [40]. In addition, as demonstrated by in silico studies, BaCf3 interacts with GLUT1 (Glucose transporter 1), which is overexpressed in many types of cancer, including lung cancer, and with the kinase domain of the MET receptor (MET Proto-Oncogene, Receptor Tyrosine Kinase), whose overexpression is detected in 25–50% of NSCLC patients. All this indicates that BaCf3 is worth considering in further research as a potential drug against lung cancer.

The azurin protein produced by *Pseudomonas aeruginosa* has pro-apoptotic properties, and the p28 peptide itself, i.e., a derivative of azurin, located at the C-terminal part of the protein chain, has anticancer properties [41]. As indicated by Huang et al., both azurin and its peptide derivative can integrate functionally with many signaling pathways involved in carcinogenesis, e.g., by stabilizing the p53 protein or regulating the activity of tyrosine kinase signaling pathways [41]. Thus, they indicate that, as potential anticancer drugs, they do not induce resistance as molecularly targeted treatments, which must be constantly refined to target cells that have undergone clonal selection for the occurrence of genetic markers of resistance. It can also be proposed that they can be used as gene-agnostic drugs targeting tyrosine kinase domains present in many genes hyperactivated in cancer. In addition to p28, Garizo et al. indicated CT-p19LC as an inducer of lung cancer cell death [42]. They conducted research on line A549; the concentrations of both peptides from 10 to 100 µM exhibited cytotoxic activity against the cancer cells and a dose–response effect was evident [42]. Furthermore, the treatment with CT-p26 led to an approximately two- to seven-fold higher decrease in cell viability than the treatment with p28 [42].

*Bacillus subtilis* is a source of natural lipopeptides exerting pro-apoptotic and cell cycle inhibitory effects. Yin et al. proved that fengicin reduced the proliferation of lung cancer cells (95D) and showed that it inhibited tumor growth in in vivo studies [43]. Moreover, this lipopeptide was able to induce ROS production and calcium ion uptake as well as LDH release and loss of mitochondrial membrane potential [43]. The study evidenced that this compound induced apoptosis of the lung cancer cells and cell cycle arrest in the G0/G1 phase by reducing the expression of cyclin D1 and CDK4 (cyclin-dependent kinase 4). The ability to induce apoptosis was linked to the mitochondrial pathway. The researchers observed that fengicin increased the activity of caspase and the expression of the pro-apoptotic Bax protein (BCL2 Associated X, Apoptosis Regulator) and reduced the expression of the anti-apoptotic Bcl-2 protein (BCL2 Apoptosis Regulator) [40]. *Bacillus subtilis* is known to produce metabolites, e.g., fengicin, iturin, and surfactin, which are effective against lung cancer cells [44]. Shao et al. purified *Bacillus subtilis* NC16 metabolites and showed that metabolite extracts suppressed proliferation and migration but promoted apoptosis of NSCLC cell lines through the caspase-3/7 signaling pathway [45]. There is no information about the exact components of the purified extract that act in this way. It is necessary to identify and check each fraction of purified metabolites, as a mixture of metabolites comprising bacteriocins/peptides may act synergistically against lung cancer cells. Routhu et al. isolated an exopolymeric biosurfactant composed of lipoheptapeptides from *Bacillus atrophaeus* AKLSR [46]. The identified cyclic lipopolypeptide (CLP) variants forming one major polymeric lipopeptide were shown to cause lung cancer cell death but were not toxic to normal cells. It induced apoptosis, cell cycle arrest of A549 cells, ROS accumulation, nuclear fragmentation, and cell death [46].

**Table 1:** Examples of compounds of bacterial origin showing anticancer abilities against lung cancer cells.

Anti-NSCLC Agent	Type of Substance	Source	Anticancer Action	Citation
Cinerubin B	anthracycline antibiotic	*Streptomyces* sp. *CMAA 1527*	to be studied	[32]
Actinomycin V	antibiotic	*Streptomyces* sp. *CMAA 1653*	cytotoxicity, induction of apoptosis, and EMT transition blockade	[32,33,34]
Enterocin 12a	bacteriocin	*Enterococcus faecium 12a*	inhibition of cancer cell growth	[35]
Colicin N	bacteriocin	*Escherichia coli*	cytotoxicity and induction of apoptosis	[36,37]
Nisin ZP	peptide	*Lactococcus lactis*	inhibition of proliferation and induction of apoptosis	[38]
Laterosporulin10	bacteriocin/peptide	*Brevibacillus* sp. *strain SKDU10*	cytotoxicity, apoptosis induction, and necrotic death induction	[39]
BaCf3	bacteriocin/peptide	*Bacillus amyloliquefaciens*	proliferation inhibition	[40]
Azurin	protein	*Pseudomonas aeruginosa*	pro-apoptotic properties	[41,42]
p28	peptide	Azurin from *Pseudomonas aeruginosa*	pro-apoptotic properties	[41]
CT-p26	peptide	Azurin from *Pseudomonas aeruginosa*	cytotoxic activity, decrease in cell viability, pro-apoptotic properties	[41]
Fengicin	lipopeptide	*Bacillus subtilis*	proliferation and tumor growth inhibition, and induction of apoptosis	[44]
Polymeric lipopeptides formed by cyclic lipopolypeptides	lipopeptides	*Bacillus atrophaeus* AKLSR	induction of apoptosis, cell cycle arrest, ROS accumulation, nuclear fragmentation, and cell death	[46]

## 4. Secondary Metabolites Synthesized by Endophytes and Marine-Derived Fungi with Lung Cancer Treatment Potential

Fungi constitute a very large group of microorganisms synthesizing secondary metabolites with different anticancer properties, including their activity against lung cancer. Previous model studies on the possibility of the application of fungal metabolites in the treatment of cancer investigated edible mushrooms used in Chinese medicine [47,48]. In this case, the anticancer properties of fungal metabolites were tested using extracts (aqueous, ethanol, and methanol) or chemical compounds obtained from fungal cultures [47,48,49]. Usually, the cytotoxicity and antiproliferative properties of lung cancer cell lines [49] and the level of gene expression responsible for tumor growth were estimated in fungal extracts [48].

Fungi are a source of a wide range of bioactive secondary metabolites, but in terms of practical application, importance is assigned to bioactive metabolites synthesized by non-toxigenic fungi (marine-derived and endophytic). Table 2 shows anticancer activities of secondary metabolites from endophytic and marine-derived fungi used against lung cancer. Therefore, this chapter will discuss compounds synthesized by these fungi and their potential in the treatment of lung cancer. Metabolites synthesized by endophytic and marine-derived fungi vary in terms of chemical structure. They mainly include polyketides, pyrones, chromones, isocoumarins, xanthones, phenalenones, diphenyl ethers, terpenoids, meroterpenoids, sesquiterpenoids, diterpenoids, triterpenoids, steroids, macrolides, lactones, alkaloidsazaphilones, terphenyls, cytochalasans, anthracenones, polyketide-terpene hybrids, polyketide-nonribosomal peptide hybrids, and quinones [48,50,51]. Among non-toxigenic fungi, endophytic fungi, i.e., symbiotic fungi that colonize various tissues of their host, most often plants, deserve special attention. Endophytes are an excellent source of bioactive compounds with anticancer activity, including those against lung cancer [52,53,54,55,56,57,58]. In addition to endophytic fungi, bioactive metabolites with anticancer properties against lung cancer are synthesized by marine-derived fungi [59,60,61,62] (Table 1). Paclitaxel mentioned in the introduction, i.e., the best known and most extensively studied anticancer metabolite characterized in the 1990s, is synthesized by the endophytic fungus *Taxomyces andreanae* [56]. Currently known under the brand name Taxol^®^, it is widely used in anticancer treatment [63]. In 1999, the Food and Drug Administration (FDA) approved the use of Taxol^®^ for the treatment of non–small cell lung cancer (NSCLC) [64]. This tubulin-binding agent is used for the treatment of small cell lung cancer (SCLC) as well [65]. 

One of the main mechanisms of action of anticancer fungal metabolites is the inhibition of the transcription factor, i.e., the NF kappa beta factor (nuclear factor kappa-light-chain-enhancer of activated B cells) [50]. NF-κB is a key player in inflammation, cancer development, and progression. Moreover, NF-κB stimulates cell proliferation, prevents apoptosis, and promotes tumor angiogenesis and metastasis [66].

The anticancer activity of fungal metabolites against lung cancer was usually tested in A549, Calu-3 [60] (adenocarcinoma lung cancer), HL251 (human lung cancer), 95-D (lung cancer cells), NCI-H187 (small cell lung cancer), H522-T1, NCI-H460, NCI-H1650, and NCI-H1975 (non-small cell lung cancer) human cell lines (Table 1). The fungal secondary metabolites applied to these cells exerted different effects, as shown by the wide range of IC_50_ values (from 0.003 to 100 and even higher) (Table 1). However, from the practical point of view and their potential use in the treatment of lung cancer, the most important agents are secondary metabolites synthesized by fungi for which the IC_50_ values are in the range of 0.002–3 µM depending on the type of cell line; these values are similar to the IC_50_ of the currently used anticancer drugs serving as positive controls in experiments, i.e., adriamycin, doxorubicin, epirubicin, and afatinib [56,67,68,69,70,71,72,73,74]. The experiments carried out so far on adenocarcinoma lung cancer cell line A549 have shown the highest cytotoxic activities of Alternarior 9-methyl ether (IC_50_ = 2.26 µM), Altertoxin II (IC_50_ = 1.15 µM), Versixantone L (IC_50_ = 1.60 µM), Asperterphenyllin G (IC_50_ = 0.40 µM), Prenylcandidusin G (IC_50_ = 2.80 µM), Prenylterphenyllin H (IC_50_ = 0.40 µM), Aspergillusone D (IC_50_ = 0.20 µM), Malformin E (IC_50_ = 2.42 µM), 21-*epi*-ophiobolin O (IC_50_ = 0.60 µM), Ophiobolin O (IC_50_ = 2.40 µM), 6-Formamide Chetomin (IC_50_ = 0.027 µM), Hispidulone B (IC_50_ = 2.71 µM), Sinopestalotiollide D (IC_50_ = 2.14 µM), 12′-hydroxyroridin E (IC_50_ = 2.08 µM), and Xanthocillins X (IC_50_ = 0.38 µM) synthesized by *Alternaria* sp. LV52, *Aspergillus versicolor HDN1009*, *Aspergillus candidatus* LDJ-5, *Aspergillus clavatus* L, *Aspergillus tamari FR02*, *Aspergillus ustus* 094102, *Chaetomium* sp. M336, *Chaetosphaeronema hispidulum*, *Pestalotiopsis palmarum*, *Myrothecium roridum* E-1069, and *Penicillium chrysogenum* CCTCC M 202001, respectively [69,75,76,77,78,79,80,81,82,83]. A previous study reported higher anticancer activity against lung cancer cells NCI-H460 exhibited by the following fungal secondary metabolites: (−)-(10E,15S)-4,6-dichloro-10(11)-dehydrocurvularin (IC_50_ = 1.45 µM), Chaunolidone A (IC_50_ = 0.09 µM), Beauvericin (IC_50_ = 1.41 µM), Bikaverin (IC_50_ = 0.43 µM), 3-*epi*-Waol A (IC_50_ = 1.00 µM), Epiroridin E (IC_50_ = 0.03 µM), Mytoxin B (IC_50_ = 0.07 µM), and Epiroridine acid (IC_50_ = 0.36 µM) synthesized by *Alternaria* sp. AST0039, *Chaunopycnis* sp. CMB-MF028, *Fusarium oxysporum* EPH2RAA, *Fusarium oxysporum* CECIS, *Libertella blepharis* F2644, and *Myrthecim roridum* A553 [61,79,84,85,86]. In the case of non-small cell lung cancer cell line NCI-H1975, secondary fungal metabolites Rhytidenone H (IC_50_ = 0.25 µM) and Rhytidenone F (IC_50_ = 1.17 µM) produced by *Rhytidhysteron rufulum* AS21B showed the highest anticancer activity [74] (Table 1). The most effective secondary fungal metabolites, i.e., (−)-(10E,15S)-4,6-dichloro-10(11)-dehydrocurvularin (*Alternaria* sp. AST0039), Chaunolidone A (*Chaunopycnis* sp. CMB-MF028), Beauvericin (*Fusarium oxysporum* EPH2RAA), Bikaverin (*Fusarium oxysporum* CECIS), 3-*epi*-Waol A (*Libertella blepharis* F2644), Epiroridin E, Mytoxin B, Epiroridine acid (*Myrthecim roridum* A553), Mycoleptodiscin B (*Mycoleptodiscus* spp. F0194), Brocazines F (*Penicillium brocae* MA-231), and Cytochalasin C, D, Q (*Xylaria* spp. NC1214), against non-small cell lung cancer HCI-H460 were characterized by the following IC_50_ values: 1.45, 0.09, 1.41, 0.43, 1.00, 0.003, 0.007, 0.36, 0.66, 0.89, 0.22, 1.06, and 1.51 µM, respectively [61,79,84,85,86,87,88,89]. Chinworrungsee et al., 2008 [90,91] indicated high cytotoxic activity of Brefeldin A, 8-deoxy-trichothecin, 7-hydroxytrichodermol (IC_50_ = 0.11; 1.48 and 1.73 µM) from strain KLAR 5 (*Hypocreales*) and Xanthoquinodin B9 (IC_50_ = 0.98 µM) from *Chaetomium globosum* 7s-1 against small cell lung cancer HCl-H187. Moreover, the cytotoxicity of Mycoleptodiscin B synthesized by *Mycoleptodiscus* spp. F0194 against non-small cell lung cancer H522-T1 was characterized by IC_50_ = 0.63 µM [87].

**Table 2:** Anticancer activities of secondary metabolites from endophytic and marine-derived fungi against lung cancer.

Fungal Strains	Compounds	IC_50_ Values (µM)	Lung Cancer Cell Lines	References
*Alternaria* sp. LV52	Alternarior 9-methyl ether	2.26	A549	[75]
	Altertoxin II	1.15		
*Aspergillus versicolor HDN1009*	Versixantone G	17.80		[62]
	Versixantone H	19.20		
	Versixantone L	1.60		
	Versixantone M	11.70		
*Aspergillus candidatus* LDJ-5	Asperterphenyllin G	0.40		[92]
	Prenylcandidusin E	19.10		[93]
	Prenylcandidusin G	2.80		
	Prenylterphenyllin F	10.20		
	Prenylterphenyllin G	16.30		
	Prenylterphenyllin H	0.40		
	Prenylterphenyllin I	14.80		
	Prenylterphenyllin J	7.60		
*Aspergillus clavatus* L	Aspergillusone C	41.90		[81]
	Aspergillusone D	0.20		
*Aspergillus micronesiensis* *MH938722*	Cyschalasin B	16.79		[94]
* Aspergillus oryzae* *KM999948*	Oryzaein B	4.20		[95]
	Oryzaein A	6.50		
	Oryzaein C	6.80		
*Aspergillus tamarii* *FR02*	Malformin E	2.42		[76]
*Aspergillus ustus* 094102	21-*epi*-ophiobolin O	0.60		[77]
	Ophiobolin O	2.40		
	21-deoxyophiobolin K	15.10		
	Ophiobolin Q	33.80		
	Ophiobolin X; 21,21-*O*-dihydro6-epi-ophiobolin G	>50		
*Aspergillus* sp. SCSIO41407	Flavoglaucin	22.20		[96]
*Aspergillus fumigates* 2011041507–5	Alkaloids fumiquinazoline J	26.90		[97]
	Fumiquinazoline C	33.40		
	Trypacidin	31.00		
*Aspergillus versicolor* F210	Proversilin C	15.00		[98]
	Proversilin E	28.40		
	Proversilin A	>40		
	Proversilin B	>40		
	Proversilin D	>40		
*Cordyceps taii*	Deacetylcytochalasin C	13.62		[99]
	Zygosporin D	16.72		
	Cytochalasins 2	17.13		
	Cytochalasins 3	19.92		
	Cytochalasins 1	32.28		
*Chaetomium* *globosum* kz-19	Penochalasin J	14.90		[70]
	Phychaetoglobin C	22.30		
	Phychaetoglobin D	13.70		
	Chaetoglobosin C	7.60		
	Chaetoglobosin E	12.30		
	Chaetoglobosin G	7.30		
	Chaetoglobosin V	11.00		
	Chaetoglobosin J	13.40		
*Chaetomium* sp. M336	6-Formamide Chetomin	0.027		[78]
*Chaetosphaeronema* *hispidulum*	Hispidulone B	2.71		[83]
*Emericella* sp. TJ29	Emeridone D	11.33		[100]
*Pestalotiopsis palmarum*	Sinopestalotiollide D	2.14		[82]
	Sinopestalotiollide A	31.29		
	Sinopestalotiollide C	36.13		
	Sinopestalotiollide B	44.89		
	2*H*-pyran-2-one	47.82		
*Phoma* sp. SYSU-SK-7	Colletotric A	37.73		[101]
	Colletotric A	20.75		
*Hypocrea lixii* R-18	Cajanol	20.50–32.80		[102]
*Eupenicillium* sp. HJ002	Penicilindole A	5.50		[72]
	Penicilindole B	18.60		
*Fusarium* sp. 2ST2	Fusarisetins E	8.70		[103]
	Fusarisetins F	4.30		
*Fusarium oxysporum* GU250648	Beauvericin	10.40		[104]
*Lasiodiplodia theobromae* ZJ-HQ1	Chloropreussomerin A	8.50		[68]
	Chloropreussomerin B	8.90		
	Preussomerin A	40.20		
	Preussomerin D	6.60		
	Preussomerin F	7.70		
	Preussomerin G	6.20		
	Preussomerin H	9.40		
	Preussomerin K	5.40		
	Preussomerin M	36.10		
*Myrothecium roridum* E-1069	12′-hydroxyroridin E	2.08		[105]
	Myrotoxin A	3.56		
	Mytoxin C	33.00		
	2′,3′-epoxymyrothecine A	36.45		
	Vertisporin	47.00		
	14′-hydroxymytoxin B	49.00		
	13′,14′-hydroxymytoxin B	53.00		
	Roridin E	55.00		
	Myrothecine A	95.00		
*Penicillium chrysogenum* V11	Penochalasin I	16.13		[67]
	Penochalasin J	35.93		
	Penochalasin K	8.73		[106]
	Chaetoglobosin A	6.56		[67]
	Chaetoglobosin C	17.82		
	Chaetoglobosin E	36.63		
	Chaetoglobosin F	27.72		
*Penicillium chrysogenum* AD-1540	Chryxanthone A	41.70		[107]
	Chryxanthone B	20.40		
*Penicillium chrysogenum* CCTCC M 2020019	Xanthocillins X	0.38		[80]
	Xanthocillins Y1	5.04		
	2-aminophenoxazin-3-one	25.60		
	Chrysomamide; *N*-[2-trans-(4-hydroxyphenyl)ethenyl]formamide;	42.87		
	*N*-acetylquestiomycin A	52.61		
*Penicillium chrysogenum*	Penichryfurans A	>100		[108]
	Penichryfurans B	87.90		
*Penicillium polonicum* TY12	Polonidine A	15.00 *		[109]
*Penicillium* sp. sh18	Isopenicin A	37.06		[110]
	Isopenicin B	>40		
	Isopenicin C	>40		
*Preussia similis*	Preussilide C	22.90		[111]
	Preussilide E	41.20		
	Preussilide D	47.90		
	Preussilide A	60.30		
	Preussilide B	70.30		
*Rhizopycnis vagum Nitaf22*	Rhizopycnin C	25.50		[112]
*Trichoderma citrinoviride*	Bislongiquinolide	11.00		[113]
	Dihydrotrichodimerol	33.00		
*Trichoderma reesei* HN-2016-018	24-hydroxy-trichodimerol	5.10		[114]
*Mucor irregularis* QEN-189	Penitrem A	8.40		[115]
	Penitrem C	8.00		
	Penitrem F	8.20		
	Rhizovarin A	11.50		
	Rhizovarin B	6.30		
	Rhizovarin E	9.20		
	3b-hydroxy-4b-desoxypaxilline	4.60		
Dichotomomyces sp. L-8	(3*R*,6*R*)-bassiatin	14.54	Calu-3	[60]
*Pestalotiopsis* m. EF01	Paclitaxel (=taxol)	0.50	HL251	[116]
*Alternaria* sp. A744	Alterperylenol	5.47	H460	[117]
	Altertoxin II	9.67		
	6-epi-stemphytriol	43.31		
	Isobenzofuranone A; Indandione B; Isosclerone; 2,4,8-trihydroxy-1-tetralone; 3,4-dihydro-3,4,8-trihydroxy-1[2*H*]-naphthalenone; 6-hydroxyisosclerone; cis-4-hydroxyscytalone; alternariol-4-methyl ether; Dihydroalterperylenol; alterperylenol	>100		
*Alternaria* sp. AST0039	(−)-(10*E*,15*S*)-4,6-dichloro-10(11)-dehydrocurvularin	1.45		[84]
	(−)-(10*E*,15*S*)-6-chloro-10(11)-dehydrocurvularin	3.57		
*Aspergillus* sp. HN15-15D	Aspergisocoumrin A	21.53		[71]
*Aspergillus oryzea*	Paclitaxel (=taxol)	50.00 *		[118]
*Aspergillus fumigates* 2011041507–5	Alkaloids pyripyropene A	38.30		[97]
	Trypacidin	33.80		
*Bipolaris sorokiniana* A606	Cochlioquinone H	15.40		[119]
	Cochlioquinone G	26.90		
	Isocochlioquinone E	31.10		
	Isocochlioquinone D	42.60		
*Cerrena* sp. A593	Cerrenin D	29.67		[120]
*Chaunopycnis* sp. CMB-MF028	Chaunolidone A	0.09		[61]
*Chaetomium globosum*	Globosumone A	6.50		[121]
	Globosumone B	24.80		
*Cytospora rhizophorae* A761	Cytorhizin B	32.80		[122]
	Cytorhizin C	54.70		
*Didymella* sp. CYSK-4	Ascomylactam A	4.40		[123]
	Ascomylactam B	13.00		
	Ascomylactam C	4.40		
	Phomapyrrolidone C	12.00		
	Pyrrolidone A	28.00		
*Fusarium oxysporum* EPH2RAA	Beauvericin	1.41		[85]
*Fusarium oxysporum* CECIS	Bikaverin	0.43		
*Libertella blepharis* F2644	3-*epi*-Waol A	1.00		[86]
*Myrthecim roridum* A553	Epiroridin E	0.003		[79]
	Mytoxin B	0.007		
	Epiroridine acid	0.36		
*Mycoleptodiscus* spp. F0194	Mycoleptodiscin B	0.66		[87]
*Penicillium brocae* MA-231	Brocazines F	0.89		[88]
*Pestalotiopsis flavidula*	2′-aminodechlorogeodoxin	16.47		[124]
	2′-aminodechloromaldoxin	18.63		
*Phyllosticta spinarum*	Tauranin	4.30		[125]
strain PM0651480	Ergoflavin	4.00		[126]
*Xylaria* spp. NC1214	Cytochalasin C	0.22		[89]
	Cytochalasin D	1.06		
	Cytochalasin Q	1.51		
*Cladosporium* sp. OUCMDZ-302	7-*O*-αD-ribosyl-5-hydroxy-2-propylchromone	10.00	HCI-H1975	[73]
*Aspergillus versicolor HDN1009*	Versixantone G	9.80		[62]
	Versixantone H	5.30		
	Versixantone M	3.50		
	Versixantone N	8.80		[69]
	Versixantone O	8.50		
*Rhytidhysteron rufulum* AS21B	Rhytidenone H	0.25		[74]
	Rhytidenone F	1.17		
	Rhytidenone G	7.30		
	Rhytidenone E	10.24		
	Deoxypreussomerin B; Palmarumycin CP17; 1-oxo-1,4-dihydronapthalene-4-spiro-20-naptho[400-hydroxy-100,800-de] [10,30]-dioxin; Preussomerin EG4; CJ-12,371; 4-*O*-methyl-CJ-12,371; Palmarumycin C5; Rhytidone A	>100.00		
*Pleosporales* sp. Sigrf05	Pleospyrone E	6.26	HCI-H1650	[127]
	Pleospyrone A	15.10		
	Pleospyrone D	29.60		
*Rhizopycnis* v. Nitaf22	TMC-264	3.20		[112]
*Chaetomium globosum* 7s-1	Xanthoquinodin B9	0.98	HCI-H187	[91]
*Eutypella* sp. BCC 13199	ent-4(15)-eudesmen-11-ol-1-one	11.00		[128]
	Eutypellin A	12.00		
strain KLAR 5 Hypocreales	Brefeldin A	0.11		[90]
	8-deoxy-trichothecin	1.48		
	7-hydroxytrichodermol	1.73		
	Trichothecolone	11.31		
	7-hydroxyscirpene	27.76		
*Phomopsis* spp. BCC 9789	Oblongolide Z	32.00		[129]
*Xylaria* spp. BCC 21097	Eremophilanolide 1	7.20		[130]
	Eremophilanolide 2	3.80		
	Eremophilanolide 3	5.80		
*Xylaria* cf. c. PK108	Cytochalasin D	5.95		[131]
	Ergosterol peroxide	5.81		
*Mycoleptodiscus* spp. F0194	Mycoleptodiscin B	0.63	H522-T1	[87]
*Cordyceps taii*	Deacetylcytochalasin C	3.67	95-D	[99]
	Zygosporin D	4.04		
	Cytochalasins 3	20.69		
	Cytochalasins 1	23.67		
	Cytochalasins 2	26.03		

* µg/mL.

## 5. Conclusions

The abundance of natural compounds in nature prompts research into their use in the treatment of cancers, including lung cancer. As natural compounds, peptides have great potential in this approach, and their effects on induction of apoptosis or inhibition of proliferation of cancer cells have been observed by many researchers. Peptides appear to be highly promising compounds for research into their use as anticancer drugs due to their structure, greater ease of production compared to full-length proteins, chemical properties leading to destruction of cancer cell membranes and ultimately entire cells, and the ability to penetrate the blood–brain barrier. Their abundance and ability to be modified by adding functional groups make them broadly applicable in oncological approaches, but more comprehensive research of individual peptides is required to determine their efficacy and possible toxic effects on healthy cells. Natural peptides may be adopted as targeted aptamer therapies, targeting specific cancer cell receptors. These issues leave a wide field for research, which may lead to the development of new targeted therapies based on naturally derived peptides. Moreover, the huge number of compounds produced by fungi generates the need to conduct studies in this field. As shown in this review, their IC_50_ values vary, and they may act in a dose-dependent manner. The papers cited here present in vitro investigations of model lung cancer cell lines. Future in vivo studies will be of great interest, as they may provide information about the toxicity of these compounds and their potential for implementation in clinical practice. This should be preceded by clinical trials determining the possibility of using these compounds alone or as supportive therapies for therapeutics that are currently registered for use in clinical practice.

## Data Availability

Not applicable.
